# Prevalence and Patterns of Oral Behaviors in Romanian Adults: An Exploratory Study

**DOI:** 10.3390/medicina61101857

**Published:** 2025-10-16

**Authors:** Alexandra Lavinia Vlad, Olivia Andreea Marcu, Ioana Scrobota, Ioan Andrei Țig, Raluca Ortensia Cristina Iurcov, Gabriela Ciavoi

**Affiliations:** 1 Doctoral School of Biomedical Sciences, Faculty of Medicine and Pharmacy, University of Oradea, 410087 Oradea, Romania; alvlad@uoradea.ro; 2 Department of Preclinical Disciplines, Faculty of Medicine and Pharmacy, University of Oradea, 410068 Oradea, Romania; omarcu@uoradea.ro; 3 Department of Dental Medicine, Faculty of Medicine and Pharmacy, University of Oradea, 410068 Oradea, Romania; riurcov@uoradea.ro (R.O.C.I.); gciavoi@uoradea.ro (G.C.)

**Keywords:** oral behavior checklist (OBC-21), temporomandibular disorders (TMD), oral behaviors, prevalence, observational study, risk assessment, Romanian adult population

## Abstract

*Background and Objectives:* Harmful repetitive oral behaviors impose an excessive load on the stomatognathic system. Being unconscious and involuntary, patients are often unaware of their occurrence and, consequently, of their potential consequences. We aimed to screen the Romanian population for harmful oral habits, while simultaneously emphasizing the importance of employing validated and internationally accepted diagnostic instruments for a better approach to these conditions. *Materials and Methods:* An observational, descriptive study was conducted on 459 adults, recruited through a multiregional convenience sampling from the general population in Romania. Oral behaviors were assessed using the validated Oral Behaviors Checklist (OBC-21) questionnaire. Data was analyzed using descriptive and comparative statistics, with significance set at *p* < 0.05. *Results:* The sample included 363 women (79.1%) and 96 men (20.9%), with a mean age of 33.3 years. The mean total OBC score was 22.45 ± 10.27, indicating a moderate prevalence of oral behaviors. 60.1% of participants were classified as low-risk and 39.9% as high-risk, with none in the no-risk category. The most frequently reported behaviors were sleeping positions exerting pressure on the mandible (57.7% “very often”), sustained talking (11.3%), and nocturnal bruxism (10.5%). Younger adults (20–49 years) presented significantly higher OBC scores compared to both younger extremes (18 years) and older adults (>60 years) (*p* < 0.001). No significant gender differences were observed in total OBC scores; however, unilateral chewing, sustained talking, and holding objects between the teeth were significantly more frequent among women (*p* < 0.05). *Conclusions:* This is the first study to investigate oral behaviors in a Romanian adult population. Postural and involuntary activities were the most prevalent and age influenced OBC scores, while gender differences were limited to individual behaviors. Conducting screening and implementing therapeutic interventions based on the assessed level of risk could enhance the overall management of the condition.

## 1. Introduction

A fundamental prerequisite for analyzing the significance and impact of oral habits is the precise, operational definition of the term “habit”. From a behavioral perspective, a habit represents a recurrent activity that occurs automatically, often without conscious awareness. During early childhood, repetitive motor behaviors are frequently observed, many of them being transient in nature and constituting an integral component of normal neuromuscular development. Nevertheless, some of them persist with age and others, like compulsive oral habits are developed during adulthood, impacting upon the dentoalveolar system [1]. Moreover, parafunctional oral activity was found to be one of the five factors that should be considered when evaluating temporomandibular joint disorders (TMD) [2].

The prevalence of oral habits varies significantly worldwide, depending on age, gender, socio-cultural context, and the assessment methods employed. Habits such as nail biting, thumb sucking, lip biting, and teeth grinding were found more frequently among both children and adults [3]. In studies including only adults, a higher prevalence of nail biting and bruxism was reported [4,5] and awake bruxism, one of the harmful oral habits known to be experienced during stress has been found one in four individuals [6].

Stress and oral habits were significantly correlated and it was stated that the presence of one of these conditions could represent a predisposing factor for the occurrence of the other [4,7]. Understanding the bidirectional nature of this interaction is essential for developing integrated approaches to evaluation and treatment, targeting both oral and mental health. A holistic perspective and evidence-based interventions, healthcare professionals can provide effective support to patients, contributing to the alleviation of problems associated with these behaviors and to the improvement of quality of life [4].

Although oral behaviors and their clinical implications have been extensively investigated across diverse populations worldwide, there is lack of data concerning the prevalence, distribution and patterns of these behaviors among Romanian adults. Existing studies focus primarily on pediatric or adolescent populations [8], or examine isolated behaviors such as bruxism [7], without providing a comprehensive assessment through standardized and validated instruments. Moreover, few investigations have simultaneously explored the sociodemographic determinants of oral behaviors, particularly within an Eastern European context.

The present study aims to assess the prevalence and patterns of oral behaviors in the adult Romanian population using the internationally validated Oral Behavior Checklist (OBC-21). We hypothesize that parafunctional oral behaviors are frequently encountered among Romanian adults and follow patterns similar to those reported internationally, with possible variations influenced by demographic factors such as age and gender. The primary objective is to determine the prevalence of parafunctional oral behaviors, while secondary objectives include characterizing their patterns, classifying participants according to risk level, and analyzing differences in relation to sociodemographic variables such as gender and age for a more evidence-driven prevention and management strategies.

## 2. Materials and Methods

### 2.1. Participants

The sample included 459 adults, recruited through a multiregional convenience sampling strategy from dental practices across different regions of Romania, providing a diverse subset of the general adult population. Participants were recruited either during routine dental consultations or while receiving elective (non-urgent) dental treatments. Patients presenting with acute dental emergencies were not included.

The minimum required sample size was calculated for prevalence estimation using the standard formula *n* = [Z2p(1 − *p*)]/e2. Since no national data on the prevalence of oral behaviors in the adult Romanian population were available, we used a conservative assumption of prevalence (*p* = 0.50), which maximizes variability. With a 95% confidence level (Z = 1.96) and a 5% margin of error (e = 0.05), the minimum required sample size was estimated at approximately 385 participants. According to the National Institute of Statistics of Romania [9], the adult population (15–64 years) was about 13.986 million individuals on 1 January 2024, and the finite population correction had no material impact on the estimate. The final study sample comprised 459 respondents, exceeding the minimum requirement.

Although convenience sampling may limit the generalizability of the findings, this method was considered appropriate given the exploratory aim of the study and the lack of prior national data on this topic. The inclusion of participants from multiple regions helped ensure a diverse and informative sample. Inclusion criteria were: age ≥ 18 years, the ability to understand the Romanian language and agreement expressed through informed consent. Respondents who completed the questionnaire only partially, as well as those with acute or chronic orofacial conditions (such as persistent pain, severe temporomandibular disorders, or active oral lesions) that could influence oral behaviors were excluded. Participants with major cognitive impairments or undergoing active psychiatric treatments, which could affect the accuracy of responses, were also excluded.

All participants were informed about the purpose of the study, and participation was voluntary, anonymous, and did not involve clinical interventions.

### 2.2. Administration of the Questionnaire and Data Collection

To assess oral behaviors, the Oral Behavior Checklist (OBC) was employed, an internationally validated instrument originally included in the Research Diagnostic Criteria for Temporomandibular Disorders Axis II and subsequently integrated into the Diagnostic Criteria for Temporomandibular Disorders Axis II [10]. The OBC is a standardized behavioral screening tool comprising 21 single-choice questions (OBC-21), designed to investigate the frequency of behaviors such as teeth grinding and clenching, nail biting, cheek biting, or using the teeth to hold objects, during both wakefulness and sleep. Responses are provided on a five-point ordinal Likert scale (0–never, 1–rarely, 2–sometimes, 3–often, 4–very often), enabling a quantitative assessment of parafunctional behaviors. The total score ranges from 0 to 84 and was classified into three risk categories: “no risk” (0), “low risk” (1–24), and “high risk” (25–84).

The Oral Behavior Checklist (OBC-21) was translated into Romanian by two bi-lingual professionals with expertise in dentistry and psychology. The translation was reviewed and finalized by consensus to ensure conceptual and linguistic accuracy. Minor adjustments were made after pilot testing on a small group of adults to confirm clarity and comprehension of the items.

The OBC-21 was administered directly by the principal investigator, both the paper and the digital versions of the questionnaire were completed in the dental office. The digital version was implemented using the Google Forms platform. In both cases, the principal investigator provided instructions and assistance to ensure participants’ proper understanding and accurate completion. Participants completed the questionnaire in dental offices from different geographical regions of Romania, including both urban and rural settings, to ensure socio-demographic diversity. Data collection was carried out between October 2024 and March 2025.

All participants received clear and concise information regarding the study objectives, the voluntary nature of participation, the anonymity of responses, and the confidentiality of the data provided. Informed consent was obtained from all respondents. For the digital version, consent was recorded by selecting a dedicated checkbox located on the first page of the form, whereas for the printed version, participants provided written consent by signing a physical form. No identifying information was collected, thereby ensuring complete anonymity of participants. Data storage and processing complied with the GDPR regulations.

The study involved no clinical interventions or risks to participants and adhered to the principles of the Declaration of Helsinki. Ethical approval was obtained from the Research Ethics Committee of the Faculty of Medicine and Pharmacy, University of Oradea, in accordance with international standards for research involving human subjects (approval number: CEFMF/2, dated 30 October 2023).

All questionnaires were reviewed prior to data entry. Data entry was performed using the double-entry method. Two authors, working independently, entered the data and repeated the process after a two-week interval. The results were then compared, and discrepancies were resolved.

### 2.3. Statistical Analysis

The statistical analysis was performed using SPSS v24 software (Armonk, New York, NY, USA), a dedicated tool for statistical processing. Descriptive and comparative statistical methods were applied to the collected data. Student’s T Test was used to test significant differences in quantitative data, while for the categorical ones it was performed the Chi-square test, adjusted with the Holm-Bonferroni correction. Results were considered statistically significant at a *p*-value of 0.05, unless otherwise stated.

## 3. Results

**Figure 1:**
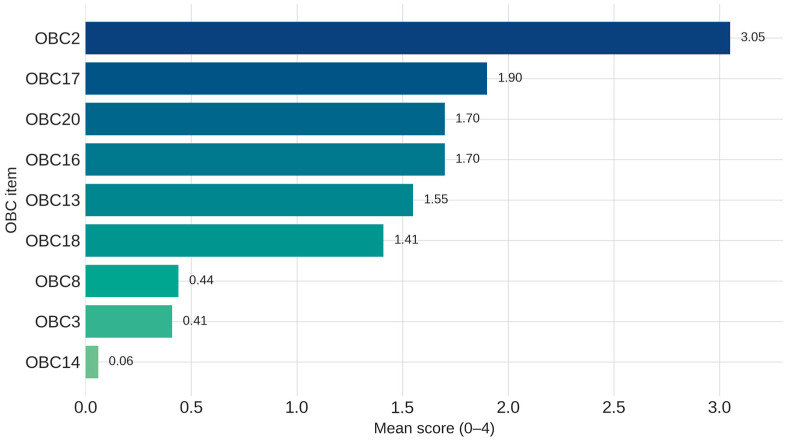
Mean scores for the most frequent and least frequent OBC items (*n* = 459).

Among the participants, 363 were women (79.08%) and 96 were men (20.92%). Age ranged from 18 to over 80 years, with a mean of 33.3 years and a median of 24.5 years (Figure 1).

The age distribution was asymmetric, showing a clear predominance of younger individuals. More than half of the sample (52.1%) fell within the 20–29 age range, followed by 22.4% in the 30–39 group and 11.3% in the 40–49 group. Only a small proportion of participants (approximately 12%) were aged 50 years or older.

The total scores exhibited a slightly asymmetric distribution (skewness = 0.67) and a slight leptokurtosis (kurtosis = 0.74), indicating a concentration of values around the mean and a moderate reduction in extreme values. This trend suggests a moderate prevalence of oral behaviors in the analyzed population, with a moderate dispersion around the mean score (Table 1).

The mean OBC score was 22.45 (SD = 10.27, Median = 21). Based on the established cutoff values, 276 participants (60.13%) were classified as low risk and 183 (39.87%) as high risk, while none of the participants fell into the no-risk category.

The mean values of the individual items ranged from 0.06 to 3.05, while the standard deviation varied between 0.80 and 1.39, indicating a moderate dispersion of scores and considerable variability in the frequency with which these behaviors were reported (Figure 1). Detailed descriptive statistics for individual items, as well as the total score, are presented in Supplementary Table S1.

The analysis of individual OBC-21 items revealed substantial variability in the frequency and intensity of reported oral behaviors.

The most frequently reported behaviors were:

- OBC2—“Sleep in a position that puts pressure on the jaw” (Mean = 3.05);
- OBC17—“Eating between meals” (Mean = 1.90);
- OBC20—“Yawning” (Mean = 1.70);
- OBC16—“Chewing food on one side only” (Mean = 1.70);
- OBC13—“Use chewing gum” (Mean = 1.55);
- OBC18—“Sustained talking” (Mean = 1.41).

In contrast, the items with the lowest mean scores included:

- OBC14—“Playing musical instrument that involves use of mouth or jaw” (Mean = 0.06);
- OBC3—“Grinding teeth together during waking hours” (Mean = 0.41);
- OBC8—“Press tongue forcibly against teeth” (Mean = 0.44).

These low mean scores indicate a limited prevalence of such behaviors in the study population.

Participants’ responses were assessed using a five-point Likert scale ranging from 0 (never) to 4 (very often). The distribution of response frequencies for each OBC-21 item revealed marked inter-item variability, reflecting differences in the expression of oral behaviors across the sample. The complete distribution of response frequencies for all items is presented in Supplementary Table S2.

The behaviors most frequently reported at the maximum frequency level (score 4–very often) included sleeping in a position that puts pressure on the jaw (OBC2), endorsed by 57.7% of participants. This was followed by sustained talking (OBC18), reported by 11.3% of participants, and nocturnal bruxism (OBC1), reported by 10.5%.

Among behaviors reported with high frequency (score 3–often), the highest proportions were observed for eating between meals (OBC17; 19.8%), chewing food on one side only (OBC16; 19.2%), and again sleeping in a position that puts pressure on the jaw (OBC2; 17.4%).

In contrast, several behaviors were rarely reported. For example, playing a musical instrument involves use of the mouth or jaw (OBC14) was reported as very often by only 0.2% of participants and as often by 0.4%. Grinding teeth together during waking hours (OBC3) was reported by 0.9% of participants for score 4 and 1.7% for score 3. Other infrequent behaviors, including hold or jut jaw forward or to the side (OBC7), holding jaw in rigid or tense position, such as to brace or protect it (OBC11), and holding, tighten, or tense muscles without clenching or bringing teeth together (OBC6), showed similarly low endorsement rates for the higher frequency categories.

The comparison of OBC total scores across age categories initially suggested a statistically significant association (χ^2^ = 536.33, *p* < 0.001) (Table 2). However, because 94.8% of the expected cell counts were below five, the assumptions for the Pearson Chi-square test were not met. In accordance with standard statistical practice, the Likelihood Ratio test was therefore applied, and the results did not confirm a significant association between age group and OBC scores (*p* = 0.987).

Descriptive examination of the score distribution indicated that moderate-to-high OBC scores (20–35) were more frequently observed among participants aged 20–49 years, whereas lower scores were typical among the youngest (18 years) and oldest (>60 years) age groups. These trends suggest that oral behaviors may be less frequent at the age extremes, although the differences were not statistically significant.

Regarding gender, the analysis revealed no statistically significant difference in total OBC scores between women (M = 22.63, SD = 10.30) and men (M = 21.75, SD = 10.19; *p* = 0.456) (Table 3). Overall, both groups displayed comparable levels of self-reported oral behaviors.

A summary of the total OBC score comparisons by gender and environment of origin is presented in Table 3, while detailed statistics for individual OBC-21 items are provided in Supplementary Table S3. *p*-values < 0.05 were considered statistically significant.

Regarding the risk level, 147 women (40.50%) were classified as high risk, while 216 (59.50%) were categorized as low risk. Among men, 70 (74.47%) were classified as low risk, whereas only 24 (25.53%) were categorized as high risk.

Statistically significant gender differences were identified for the following individual behaviors: OBC16 (“chewing food on one side only”) (*p* = 0.008); OBC18 (“sustained talking”) (*p* = 0.014); OBC19 (singing) (*p* = 0.021); and OBC7 (“hold or jut jaw forward or to the side) (*p* = 0.027). All these oral habits were more frequently observed in women.

No significant differences were found in total OBC scores between participants from urban and rural areas. Among the 326 participants from urban areas, 190 (58.26%) were classified as low risk, while 136 (41.72%) were categorized as high risk. In rural areas, the difference between the two risk levels was more pronounced, with 64.40% of participants classified as low risk and only 35.60% classified as high risk.

However, a statistically significant difference was observed regarding forceful pressing of the teeth with the tongue (*p* = 0.04) (Supplementary Table S3).

## 4. Discussion

The present study evaluated oral behaviors among 459 Romanian adults using the standardized OBC-21 questionnaire. The results revealed a moderate prevalence of oral behaviors, with postural and involuntary habits—such as sleeping in positions that exert pressure on the jaw, eating between meals, and unilateral chewing—being the most frequent. Although no significant differences emerged in total OBC scores between genders, certain individual behaviors differed significantly. Younger adults (20–49 years) displayed slightly higher scores, suggesting a greater behavioral intensity within this age range.

The study sample (*n* = 459) enabled exploratory analyses of behavioral distribution by demographic factors. While the predominance of women and younger participants limits external generalizability, it mirrors patterns observed in self-reported health surveys, where these groups typically demonstrate higher participation rates. This enhances the study’s internal validity and aligns with similar international research [11,12,13].

The OBC-21 utilized in this study was initially developed by Ohrbach and colleagues to evaluate oral behaviors during wakefulness and was subsequently adapted into its current form [14]. A study conducted by van der Meulen et al. (2014) reported significant correlations between OBC scores and facial pain intensity, supporting its construct validity [15]. Additionally, the authors reported adequate internal consistency and an enhanced ability to distinguish between voluntary and involuntary behaviors, thereby recommending it as a reliable screening tool.

The OBC-21, developed by Ohrbach and colleagues to evaluate daytime oral behaviors, has demonstrated good reliability and construct validity [14,15]. The Romanian version used in this study, translated by the principal investigator, included minimal linguistic adaptations for clarity. Although not formally validated, its use was meth-odologically justified in this exploratory context, given the international acceptance of the instrument and its integration into TMD assessment protocols [16].

Beyond the validity of the instrument itself, the administration of the questionnaire was carefully planned to minimize sampling biases. To maximize participation and enhance the representativeness of our sample, we administered the Oral Behavior Checklist (OBC-21) using both traditional paper-based methods and an online format. This dual approach allowed us to reach participants with varying preferences and accessibility. Additionally, we employed a multiregional recruitment strategy, with the principal investigator visiting multiple regions across Romania to engage participants directly and collaborate with other clinicians in different areas. This approach aimed to capture a broader geographic distribution, ensuring that the sample reflected diverse adult populations and regional contexts within the country. While convenience sampling inherently carries some limitations regarding representativeness, these efforts helped mitigate potential biases and strengthened the descriptive value of the study.

This strategy is methodologically supported by the principles of the Tailored Design Method, which recommend adapting the data collection process to standardize the respondent experience and maximize response rates, while maintaining the quality of the collected data [17].

Overall, the distribution of total OBC-21 scores indicates moderate behavioral variability, with a tendency toward higher frequencies among younger adults. This pattern may reflect psychosocial and lifestyle factors associated with active life stages and aligns with previous findings that highlight age as a determinant of oral behavioral expression [13].

These findings are consistent with observations reported by other researchers who identified a higher prevalence of oral behaviors among young adults (18–29 years). Their study, based on 1424 clinical cases, classified 43.3% of participants as high-risk (OBC score > 24), a proportion slightly above that observed in our sample (39.9%) [13]. The difference likely reflects contextual variations: the present study included predominantly asymptomatic individuals, whereas the clinical population examined by Reda et al. comprised subjects more susceptible to stomatognathic dysfunctions. This under-scores the importance of considering population characteristics when interpreting prevalence data.

At the population level, oral behaviors appear to be common even in the absence of pain or evident dysfunction [18]. This highlights the importance of behavioral screening for early prevention, particularly among individuals at increased risk of temporomandibular disorders.

From a clinical standpoint, the score distribution suggests a relatively homogeneous behavioral pattern, with a small subgroup displaying intense and potentially persistent oral behaviors. Identifying this subgroup is essential for early intervention and risk stratification [19,20]. The variability observed in OBC-21 scores reflects the multifactorial etiology of these behaviors, influenced by psychological, lifestyle, and demographic factors.

Although total scores did not differ significantly between genders, several individual behaviors—including unilateral chewing, sustained talking, and object biting—were reported more frequently by women. This aligns with previous findings suggesting that women may display greater behavioral reactivity to stress and heightened somatic awareness, potentially mediated by personality traits and coping strategies [21,22]. Conversely, the absence of significant gender differences in total OBC scores contrasts with findings from an Italian clinical study reporting higher female scores [13]. These discrepancies likely arise from contextual differences between community-based and clinical samples and emphasize the need for nuanced interpretation of gender-related pat-terns in oral behaviors.

Recent studies indicate that many adults exhibit oral behaviors—often unconsciously—that can contribute to dental wear, occlusal imbalance, and masticatory muscle overload, even in the absence of evident symptoms [5,23]. The involuntary nature of such actions, including jaw clenching, unilateral chewing, or postural habits, makes them difficult to detect and control in everyday life.

These behaviors tend to manifest selectively, with nocturnal and postural habits being more frequently reported than consciously controlled activities, suggesting only partial awareness of the parafunctional phenomenon [13]. In the present study, the most frequent behaviors—side sleeping and unilateral chewing—are consistent with previous literature identifying similar trends in young adults [24,25]. Side sleeping, in particular, has been associated with increased temporomandibular joint (TMJ) loading, pain, and reduced mouth opening, emphasizing its potential clinical impact [25].

Unilateral chewing and teeth clenching have also been linked to joint sounds and muscular tension [24]. Their repetitive, often unnoticed occurrence highlights the importance of early identification through both self-report tools and clinical evaluation.

Sustained talking (OBC18) may also reflect overuse of the stomatognathic system in daily activities. Professions involving prolonged speaking or oral exertion—such as teaching or playing wind instruments—can exacerbate muscle fatigue and contribute to functional imbalance, particularly under psychological stress, which is known to increase masticatory muscle activity [21].

These findings reinforce the clinical relevance of oral behaviors observed in the current sample, highlighting their potential etiopathogenetic role in the development of stomatognathic dysfunctions-even in the absence of a formal clinical diagnosis. Awareness and early education, particularly regarding sleep and postural habits, should be integrated into preventive and diagnostic protocols to mitigate long-term functional risks.

Another frequently reported behavior was eating between meals, a potential indicator of imbalances in dietary patterns or compensatory behaviors in response to stress. Although seemingly benign, this type of repetitive activity may contribute to overloading the masticatory muscles and the temporomandibular joint, particularly in the cumulative context of other parafunctional habits.

Nocturnal bruxism (OBC1), though less prevalent, remains clinically relevant. As an involuntary behavior, it warrants further evaluation through objective diagnostic methods or correlation with clinical signs such as dental wear and muscle tenderness [26].

Taken together, these findings suggest that even mild and seemingly harmless oral behaviors may exert a cumulative effect on the stomatognathic system. Their association with psychosocial stress underscores the need for systematic behavioral assessment using standardized tools such as the OBC-21, which should be integrated into preventive and diagnostic practice.

Future research should adopt longitudinal designs to track behavioral dynamics over time and identify predictive factors. Combining validated self-report instruments with objective measures (e.g., electromyography, occlusal analysis, or TMJ assessments) could yield a more comprehensive understanding of the relationship between oral behaviors and stomatognathic dysfunctions.

Moreover, the psychological dimension of these behaviors warrants further exploration. Emerging evidence links chronic stress and anxiety to increased orofacial activity [19,27,28]. Future studies integrating the OBC-21 with standardized psychological screening tools (e.g., GAD-7, PHQ-9) could clarify these bidirectional relationships and support the development of interdisciplinary prevention and intervention strategies.

Relevant and promising future research involves exploring the relationship between oral behaviors and mental health, particularly symptoms of depression and anxiety. Emerging evidence links chronic stress and anxiety to increased orofacial activity [19,27,28]. Future studies integrating the OBC-21 with standardized psychological screening tools (e.g., GAD-7, PHQ-9) could clarify these bidirectional relationships and support the development of integrated preventive interventions.

Overall, the present findings offer a relevant framework for understanding behavioral patterns in the general population and highlight the need for further analytical and experimental studies. Future research should focus on identifying causal mechanisms and evaluating the effectiveness of preventive interventions through longitudinal or randomized controlled designs. Such efforts would strengthen the evidence base for behavioral risk assessment and contribute to developing tailored strategies for adult oral health in Romania.

The findings of this study have important implications for public health and preventive dentistry in Romania. The moderate prevalence of oral behaviors observed in the general population, even in the absence of overt temporomandibular dysfunction, underscores the need to incorporate behavioral assessment into routine dental care. Early identification of maladaptive oral habits can facilitate timely counseling, reduce the risk of chronic orofacial pain, and improve long-term oral function. Furthermore, public health strategies should prioritize patient education on stress management, posture, and sleep hygiene, as these are modifiable determinants of oral behaviors. Implementing national or regional screening initiatives using standardized instruments such as the OBC-21 could strengthen preventive programs and enhance understanding of behavioral risk factors in oral health across Romania.

The classification of participants into low- and high-risk groups based on OBC-21 scores offers valuable insight into the spectrum of behavioral vulnerability within the general population. This differentiation is not merely statistical but holds clinical relevance, as it may guide individualized preventive and therapeutic approaches in dental practice.

For individuals categorized as low risk, the dentist’s role should primarily focus on preventive counseling and behavioral reinforcement. These patients typically exhibit mild or occasional oral habits that may not currently cause dysfunction but could become problematic over time. Providing targeted education on posture, stress management, and awareness of oral behaviors—alongside routine monitoring during regular dental visits—can help prevent the escalation of such habits and promote long-term functional stability.

In contrast, patients identified as high risk require a more comprehensive and proactive approach. Dentists should conduct detailed functional assessments of the temporomandibular system, evaluate potential muscle or joint involvement, and explore psychosocial factors contributing to these behaviors. Early interventions, including behavioral modification strategies, physiotherapy, or interdisciplinary collaboration with mental health professionals, may be indicated to address underlying stress-related mechanisms. This individualized, risk-based management can enhance the early detection of temporomandibular dysfunction and reduce its long-term clinical impact.

In summary, the present findings emphasize that oral behaviors are highly prevalent among adults, even in the absence of temporomandibular disorders. Integrating behavioral screening and counseling into routine dental practice represents a key step toward early detection, prevention, and improved long-term functional outcomes.

### Study Limitations

The use of a multiregional convenience sampling strategy does not ensure full representativeness of the Romanian adult population and may introduce selection bias. Therefore, the results should be interpreted as exploratory.

A second limitation pertains to the unbalanced structure of the sample, characterized by a predominance of women and younger participants. This demographic profile, while common in self-reported health surveys, may limit the external validity of the results. Consequently, future large-scale studies should encompass diverse regions of Romania reflect the true distribution of oral behaviors within the Romanian population.

Also, the questionnaire employed (OBC-21) was administered in a Romanian-translated version without formal psychometric validation. Although the instrument is standardized and internationally recognized, the absence of rigorous cultural adaptation in the Romanian context remains an important aspect that should be addressed in future research.

The study also relied exclusively on self-reported data, which may be subject to recall bias, as participants might not accurately remember or may underreport the frequency of certain behaviors. Social desirability bias cannot be excluded either, as some participants might have minimized the occurrence of behaviors perceived as undesirable or socially inappropriate [29]. Moreover, the absence of clinical confirmation represents a limitation of the present research. Future studies should correlate OBC-21 scores with clinical examinations to validate the accuracy of self-reported data and provide a more comprehensive understanding of the clinical significance of these behaviors. Nevertheless, the anonymous nature of data collection and the use of a standardized, validated tool partially mitigate these risks.

Finally, an additional limitation concerns the assessment of mental health. This was not performed using standardized psychiatric instruments. Instead, exclusion relied on self-reported information provided in the medical history form completed at the first dental visit, where participants disclosed ongoing treatments or medical conditions, including psychiatric care. While this approach offered a practical and ethically acceptable screening method, it does not replace a structured mental health evaluation and may limit the precision of participant selection.

A strength of the present study is that deleterious oral habits have not previously been investigated in a Romanian population, which provides originality and scientific relevance to the research. In addition, the analysis was conducted on a statistically significant number participants that supports the validity of results and confers international relevance. Furthermore, the use of an internationally validated assessment tool strengthens the methodological robustness of the study.

## 5. Conclusions

The present study investigated the prevalence and patterns of oral behaviors among the general adult population in Romania, utilizing the OBC-21 questionnaire. The findings indicate a moderate expression of these behaviors, with interindividual variability and higher scores observed among young adults (20–49 years).

These findings underscore that oral behaviors are common even among asymptomatic adults, reinforcing their relevance as early behavioral markers of functional risk and as potential targets for preventive and educational interventions.

Although the total score did not differ significantly between genders, the analysis of individual items revealed relevant differences, suggesting gender-related influences. The most frequently reported behaviors were postural, alimentary, and those associated with unconscious diurnal activities.

The heterogeneity of scores and the presence of these behaviors even in the absence of clinical symptoms underscore the relevance of behavioral screening and self-monitoring, particularly within at-risk groups. The results also highlight the importance of employing standardized instruments such as the OBC-21 in both clinical assessment and research on parafunctional behaviors.

Overall, oral behaviors appear to represent a complex, multifactorial phenomenon that requires integrated approaches combining screening, education, and prevention. Incorporating behavioral assessment into clinical protocols—even in the absence of TMD symptoms—may enhance early detection, prophylaxis, and management of related dysfunctions.

By providing population-level normative data for Romanian adults, this study fills a significant regional knowledge gap and contributes novel epidemiological evidence supporting the integration of behavioral evaluation into routine dental and public health practice. These insights also emphasize the need for interdisciplinary collaboration to strengthen behavioral awareness and early intervention at both clinical and policy levels.

These data could serve as a solid foundation for future research exploring the interaction between psychological and clinical dimensions, such as stress, anxiety, or temporomandibular dysfunctions, aimed at developing personalized strategies for early prevention and intervention within oral and mental health frameworks.

## Figures and Tables

**Table 1:** Descriptive statistics for the OBC total score.

	OBC Total Score
Mean	22.4466
Median	21.0000
Standard Deviation	10.27455
Skewness	0.67
Kurtosis	0.74

**Table 2:** Pearson Chi-Square test results for the comparison of OBC scores across age groups.

Chi-Square Tests		
	Value	Asymptotic Significance (2–Sided)
Pearson Chi-Square	536.327 ^a^	0.000
Likelihood Ratio	319.400	0.987
N of Valid Cases	459	

^a^ 417 cells (94.8%) have expected count less than 5. The minimum expected count is 0.00.

**Table 3:** Summary of total OBC scores by gender and environment of origin.

Variable	Group	N	Mean	Standard Deviation	*p*-Value
Gender	women	363	22.6309	10.30107	0.456
	men	96	21.75	10.19701	
Environment of origin	urban	326	22.75	10.15	0.330
	rural	133	21.71	10.58	

## Data Availability

The original contributions presented in this study are included in the article. Further inquiries can be directed to the corresponding authors on reasonable request.

## Supplementary Materials

The following supporting information can be downloaded at: https://www.mdpi.com/article/10.3390/medicina61101857/s1, Table S1: Mean, median, and standard deviation for total score and individual items of the Oral Behavior Checklist (OBC-21); Table S2: Distribution of response frequencies (number and percentage) for each OBC-21 item (scores 0–4); Table S3: Detailed mean, median, standard deviation, and p-values for individual OBC-21 items by sex and environment of origin.

## Abbreviations

The following abbreviations are used in this manuscript:

TMJ	Temporo Mandibular Joint
OBC	Oral Behaviors Checklist
SPSS	Statistical Package for the Social Sciences
TMD	Temporo Mandibular Disorders
