# Supplementary material for: Prevalence and Patterns of Oral Behaviors in Romanian Adults: An Exploratory Study
Source: Medicina (Kaunas). 2025 Oct 16;61(10):1857. doi: 10.3390/medicina61101857 (PMC12565779; doi:10.3390/medicina61101857)
Supplement: Supplementary file 1 [file medicina-61-01857-s001.zip › Table S1.pdf]

Table S1. Mean, median, and standard deviation for total score and individual items of the Oral Behavior Checklist (OBC-21)

	OBC	OBC1	OBC2	OBC3	OBC4	OBC5	OBC6	OBC7	OBC8	OBC9	OBC10
**Mean**	22,4466	1,1002	3,0458	0,4161	0,7843	1,1569	0,6972	0,5534	0,4292	0,4183	1,0741
**Median**	21,0000	0,0000	4,0000	0,0000	0,0000	1,0000	0,0000	0,0000	0,0000	0,0000	1,0000
**Std. Deviation**	10,27455	1,38566	1,38144	0,77561	0,99084	1,14524	0,92149	0,82462	0,80453	0,80891	1,12871
	**OBC11**	**OBC12**	**OBC13**	**OBC14**	**OBC15**	**OBC16**	**OBC17**	**OBC18**	**OBC19**	**OBC20**	**OBC21**
**Mean**	0,4641	0,8301	1,5512	0,0697	1,3725	1,6993	1,8954	1,4052	0,8192	1,6993	0,9651
**Median**	0,0000	0,0000	1,0000	0,0000	1,0000	2,0000	2,0000	1,0000	1,0000	1,0000	1,0000
**Std. Deviation**	0,80413	1,09060	1,14605	0,37314	1,03571	1,26120	1,05211	1,34670	1,02798	1,02428	1,04217

Responses are expressed on an ordinal scale reflecting the frequency of each behavior during the past 30 days.
