# Supplementary material for: Prevalence and Patterns of Oral Behaviors in Romanian Adults: An Exploratory Study
Source: Medicina (Kaunas). 2025 Oct 16;61(10):1857. doi: 10.3390/medicina61101857 (PMC12565779; doi:10.3390/medicina61101857)
Supplement: Supplementary file 1 [file medicina-61-01857-s001.zip › Table S2.pdf]

Table S2. Distribution of response frequencies (number and percentage) for each OBC-21 item (scores 0–4).

	OBC	OBC1	OBC2	OBC3	OBC4	OBC5	OBC6	OBC7	OBC8	OBC9	OBC10
**Mean**	22,4466	1,1002	3,0458	0,4161	0,7843	1,1569	0,6972	0,5534	0,4292	0,4183	1,0741
**Median**	21,0000	0,0000	4,0000	0,0000	0,0000	1,0000	0,0000	0,0000	0,0000	0,0000	1,0000
**Std. Deviation**	10,27455	1,38566	1,38144	0,77561	0,99084	1,14524	0,92149	0,82462	0,80453	0,80891	1,12871
	**OBC11**	**OBC12**	**OBC13**	**OBC14**	**OBC15**	**OBC16**	**OBC17**	**OBC18**	**OBC19**	**OBC20**	**OBC21**
**Mean**	0,4641	0,8301	1,5512	0,0697	1,3725	1,6993	1,8954	1,4052	0,8192	1,6993	0,9651
**Median**	0,0000	0,0000	1,0000	0,0000	1,0000	2,0000	2,0000	1,0000	1,0000	1,0000	1,0000
**Std. Deviation**	0,80413	1,09060	1,14605	0,37314	1,03571	1,26120	1,05211	1,34670	1,02798	1,02428	1,04217

**Scale**	**OBC1**	**OBC2**	**OBC3**	**OBC4**	**OBC5**	**OBC6**	**OBC7**
**0**	239 (52.1%)	53 (11.5%)	330 (71.9%)	236 (51.4%)	159 (34.6%)	251 (54.7%)	281 (61.2%)
**1**	67 (14.6%)	24 (5.2 %)	83 (18.1%)	125 (27.2%)	160 (34.9%)	126 (27.5%)	121 (26.4%)
**2**	69 (15%)	37 (8.1%)	34 (7.4%)	69 (15%)	69 (15%)	56 (12.2%)	42 (9.2%)
**3**	36 (7.8%)	80 (17.4%)	8 (1.7%)	19 (4.1%)	51 (11.1%)	22 (4.8%)	11 (2.4%)
**4**	48 (10.5%)	265 (57.7%)	4 (0.9%)	10 (2.2%)	20 (4.4%)	4 (0.9%)	4 (0.9%)
	**OBC8**	**OBC9**	**OBC10**	**OBC11**	**OBC12**	**OBC13**	**OBC14**
**0**	326 (71%)	332 (72.3%)	183 (39.69%)	316 (68.8%)	244 (53.2%)	87 (19%)	439 (95.6%)
**1**	92 (20%)	84 (18.3%)	136 (29.6%)	91 (19.8%)	111 (24.2%)	155 (33.8%)	12 (2.6%)
**2**	23 (5%)	27 (5.9%)	79 (17.2%)	37 (8.1%)	54 (11.8%)	128 (27.9%)	5 (1.1%)
**3**	13 (2.8%)	11 (2.4%)	45 (9.8%)	12 (2.6%)	38 (8.3%)	55 (12%)	2 (0.4%)
**4**	5 (1.1%)	5 (1.1%)	16 (3.5%)	3 (0.7%)	12 (2.6%)	34 (7.4%)	1 (0.2%)
	**OBC15**	**OBC16**	**OBC17**	**OBC18**	**OBC19**	**OBC20**	**OBC21**
**0**	96 (20.9%)	93 (20.3%)	33 (7.2%)	154 (33.6%)	228 (49.7%)	32 (7.0%)	182 (39.7%)
**1**	176 (38.3%)	130 (28.3%)	145 (31.6%)	119 (25.9%)	138 (30.1%)	201 (43.8%)	170 (37%)
**2**	123 (26.8%)	103 (22.4%)	154 (33.6%)	84 (18.3%)	52 (11.3%)	132 (28.8%)	64 (13.9%)
**3**	48 (10.5%)	88 (19.2%)	91 (19.8%)	50 (10.9%)	30 (6.5%)	61 (13.3%)	27 (5.9%)
**4**	15 (3.5%)	45 (9.8%)	36 (7.8%)	52 (11.3%)	11 (2.4%)	33 (7.2%)	16 (3.5%)

Scores were reported on the Likert scale as follows: 0 = never, 1 = rarely, 2 = sometimes, 3 = often, 4 = very often.
