# Supplementary material for: Prevalence and Patterns of Oral Behaviors in Romanian Adults: An Exploratory Study
Source: Medicina (Kaunas). 2025 Oct 16;61(10):1857. doi: 10.3390/medicina61101857 (PMC12565779; doi:10.3390/medicina61101857)
Supplement: Supplementary file 1 [file medicina-61-01857-s001.zip › Table S3.pdf]

Table S3. Detailed mean, median, standard deviation, and *p*-values for individual OBC-
and environment of origin.

	SEX	N	Mean	Standard Deviation	p-value	ENVIROMENT OF ORIGIN	N	Mean
**OBC**	0	363	22,6309	10,30107	0,456	0	326	22,7454
	1	96	21,75	10,19701		1	133	21,7143
**OBC1**	0	363	1,0826	1,39407	0,598	0	326	1,1595
	1	96	1,1667	1,35853		1	133	0,9549
**OBC2**	0	363	3,0716	1,37674	0,436	0	326	3,0706
	1	96	2,9479	1,40203		1	133	2,985
**OBC3**	0	363	0,405	0,7857	0,549	0	326	0,4417
	1	96	0,4583	0,73866		1	133	0,3534
**OBC4**	0	363	0,7989	1,00595	0,54	0	326	0,8374
	1	96	0,7292	0,93448		1	133	0,6541
**OBC5**	0	363	1,124	1,16275	0,232	0	326	1,1871
	1	96	1,2813	1,07315		1	133	1,0827
**OBC6**	0	363	0,6915	0,94232	0,797	0	326	0,7239
	1	96	0,7188	0,84234		1	133	0,6316
**OBC7**	0	363	0,5096	0,79138	0,027	0	326	0,5276
	1	96	0,7188	0,92569		1	133	0,6165
**OBC8**	0	363	0,4353	0,81293	0,754	0	326	0,4785
	1	96	0,4063	0,77566		1	133	0,3083
**OBC9**	0	363	0,3912	0,79092	0,163	0	326	0,4141
	1	96	0,5208	0,87032		1	133	0,4286
**OBC10**	0	363	1,0634	1,12915	0,693	0	326	1,0552
	1	96	1,1146	1,13202		1	133	1,1203
**OBC11**	0	363	0,4683	0,825	0,825	0	326	0,4663
	1	96	0,4479	0,72358		1	133	0,4586
**OBC12**	0	363	0,8182	1,0848	0,65	0	326	0,819
	1	96	0,875	1,11686		1	133	0,8571
**OBC13**	0	363	1,5537	1,11457	0,927	0	326	1,5613
	1	96	1,5417	1,26422		1	133	1,5263
**OBC14**	0	363	0,0606	0,35127	0,377	0	326	0,0613
	1	96	0,1042	0,44672		1	133	0,0902
**OBC15**	0	363	1,3994	1,02062	0,28	0	326	1,3926
	1	96	1,2708	1,09043		1	133	1,3233
**OBC16**	0	363	1,7796	1,26182	0,008	0	326	1,6626
	1	96	1,3958	1,2181		1	133	1,7895
**OBC17**	0	363	1,9229	1,0404	0,278	0	326	1,8988
	1	96	1,7917	1,09464		1	133	1,8872
**OBC18**	0	363	1,4848	1,36922	0,014	0	326	1,4693
	1	96	1,1042	1,2181		1	133	1,2481
**OBC19**	0	363	0,876	1,04778	0,021	0	326	0,7945

	1	96	0,6042	0,92314		1	133	0,8797
**OBC20**	0	363	1,7218	1,00948	0,362	0	326	1,7239
	1	96	1,6146	1,07967		1	133	1,6391
**OBC21**	0	363	0,9725	1,00788	0,77	0	326	1
	1	96	0,9375	1,16811		1	133	0,8797

p-values < 0.05 were considered statistically significant. Gender coding: 0 = women, 1 = men; Environment coding: 0 = urban, 1 = rural.
